# CGA-ASNet: an RGB-D amodal segmentation network for restoring occluded tomato regions

**DOI:** 10.3389/fpls.2025.1664718

**Published:** 2025-09-23

**Authors:** Zhaoyang Li, Yong Yin, Zhihong Xing, Hanbing Deng

**Affiliations:** College of Information and Electrical Engineering, Shenyang Agricultural University, Shenyang, China

**Keywords:** amodal segmentation, occlusion-aware segmentation, RGB-D image segmentation, plant phenotyping, tomato, smart agriculture

## Abstract

Obtaining the complete morphology of tomato fruits under non-destructive conditions is essential for phenotype research, yet fruit occlusions often hinder deep learning-based image segmentation methods from capturing the true shape of occluded regions. This limitation reduces prediction accuracy and adversely impacts phenotype data acquisition. To overcome this challenge, we propose CGA-ASNet, an RGB-D amodal segmentation network incorporating a Contextual and Global Attention (CGA) module. A synthetic tomato dataset (Tomato-sim) was constructed using NVIDIA Isaac Sim’s Replicator Composer (ISRC) to realistically simulate tomato morphology and greenhouse environments, and the network was trained on this dataset. To evaluate generalization, CGA-ASNet was tested on both the synthetic and a separate real-world dataset. While no explicit domain adaptation techniques were adopted, diverse lighting conditions (strong, normal, and weak illumination) were simulated to implicitly reduce the domain gap, and a mean coordinate fusion algorithm was introduced to improve annotation completeness in real-world occlusion scenarios. By leveraging contextual information among feature input keys for self-attention learning, capturing global information, and expanding the receptive field, CGA-ASNet enhanced representation capacity, semantic understanding, and localization accuracy. Experimental results demonstrated that CGA-ASNet achieved an F@0.75 score of 94.2 and a mean Intersection over Union (mIoU) of 82.4% in greenhouse amodal segmentation tasks. These findings indicate that training with well-designed synthetic datasets can effectively support accurate occlusion-aware segmentation in real environments, providing a practical solution for tomato phenotyping in greenhouse conditions.

## 1 Introduction

Tomatoes are among the most widely cultivated vegetables globally, with countries such as the United States, China, and Japan extensively utilizing greenhouse cultivation methods. In recent years, the area dedicated to greenhouse tomato farming has steadily expanded. However, despite these advancements in controlled-environment agriculture, tomato harvesting remains largely dependent on manual labor, which is not only labor-intensive but also inefficient (Cámara-Zapata et al., 2019). To address these challenges, automated growth monitoring systems and intelligent harvesting machines are gradually emerging as key solutions in modern agriculture. These technologies are increasingly being adopted to mitigate labor shortages in regions that heavily rely on manual harvesting. However, fruit morphology remains indispensable in processes such as biological control and biomass detection.

Accurate information on fruit morphology is crucial for multiple aspects of agricultural management. It not only aids in determining the growth status of plants but also supports precision fertilization and irrigation decisions. Furthermore, changes in fruit morphology can serve as early indicators of pests and diseases, enabling farmers to detect issues early and take appropriate action. Automated detection systems, through continuous monitoring of plant morphology, offer higher precision and real-time feedback, thereby reducing dependency on manual labor and improving both crop yield and quality. With the rapid advancements in machine vision and deep learning, many computer vision tasks—such as image recognition (He et al., 2016; Szegedy et al., 2016), object detection (Girshick, 2015; Redmon and Farhadi, 2018; Ren et al., 2015);, and semantic segmentation (Long et al., 2015)—have enabled precise localization and shape determination of fruits based on their appearance. These techniques have been widely applied in disease detection (Dhaka et al., 2021), maturity assessment, and growth monitoring. However, these tasks typically rely on each pixel in the image corresponding to a single label. In occlusion scenarios, models can only process visible portions, leaving occluded areas unaddressed or inadequately evaluated.

Traditional computer vision algorithms, including edge-based segmentation methods (Sheng et al., 2023), struggle to handle occlusions, particularly in agriculture, where fruits grow in random positions and complex lighting conditions further complicate scene interpretation. While edge-based approaches have shown effectiveness in fruit segmentation under certain conditions, current technologies face challenges in effectively dealing with occluded fruits, resulting in lower recognition accuracy. Current technologies face challenges in effectively dealing with occluded fruits, resulting in lower recognition accuracy. This issue presents a significant barrier to the implementation of automation in agriculture, particularly in automated harvesting and growth monitoring systems, where the presence of occlusions severely impacts recognition accuracy and operational efficiency. To effectively address this problem, the occluded portions of the fruit must be accurately reconstructed.

**Figure 1:**
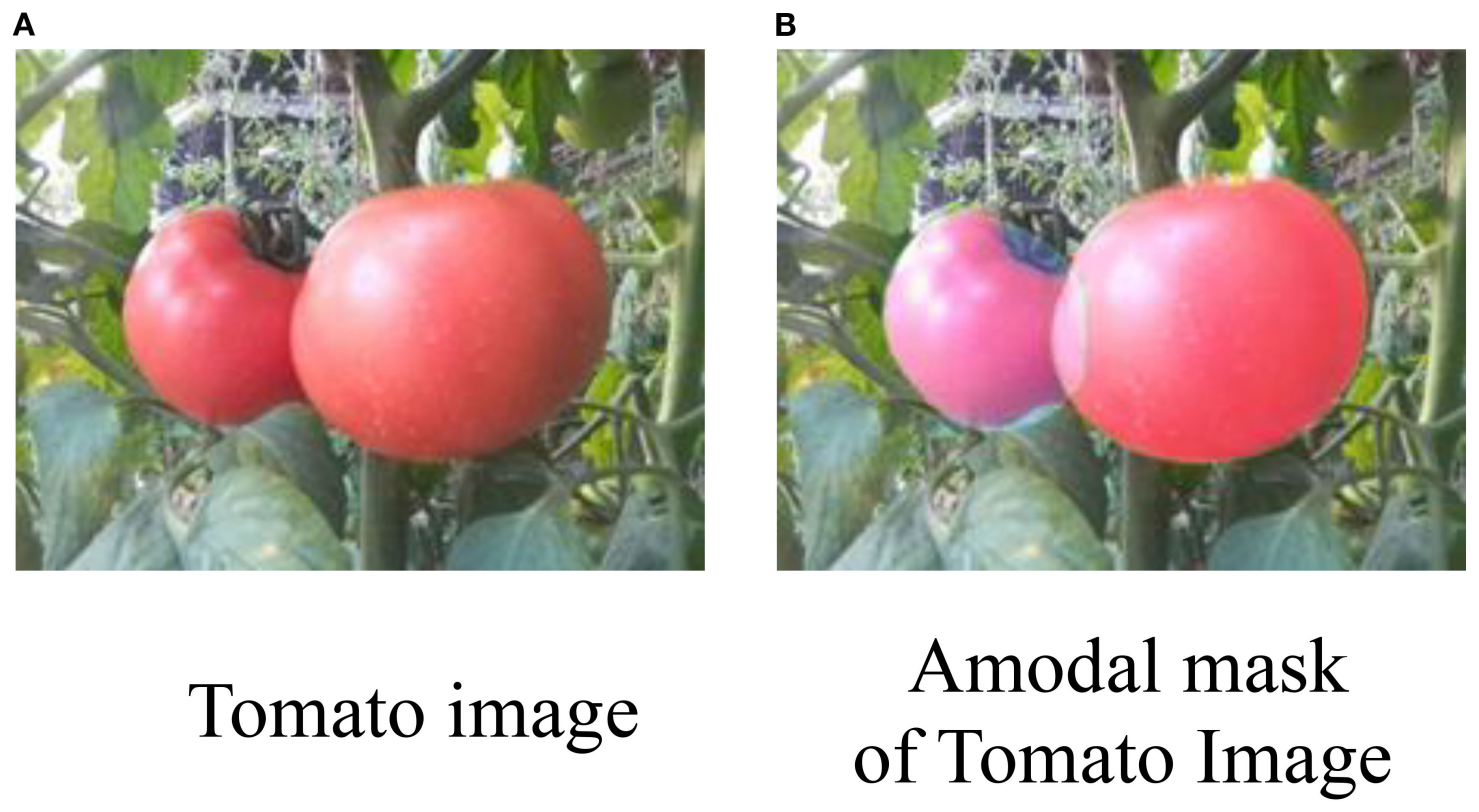
**(A)** Two ripe tomatoes on a plant with green leaves. **(B)** An amodal mask of the same tomatoes, highlighting their form against the background.

Amodal segmentation aims to infer and complete the occluded portions of objects by providing their full masks, as illustrated in **Figure 1**. Several recent studies have explored occlusion-aware perception and shape reconstruction techniques to improve fruit detection in complex agricultural environments. An occluder–occludee relational network (O2RNet) was proposed to explicitly model spatial interactions between overlapping objects and achieved state-of-the-art performance in clustered apple detection (Chu et al., 2023). A zero-shot Sim2Real reinforcement learning strategy was introduced to manipulate deformable plants and reveal hidden fruits, achieving 86.7% success without real-world fine-tuning (Subedi et al., 2025). Furthermore, a safe leaf manipulation method was proposed to improve pose and shape estimation accuracy by uncovering occluded fruits (Yao et al., 2025).

While these techniques have shown promising performance in orchard and open-field conditions, greenhouse environments present a distinct set of challenges that remain underexplored. Greenhouse-grown crops, such as tomatoes, are typically cultivated in densely packed rows with limited spacing, resulting in more frequent intra-class occlusions. The constrained physical layout, along with complex lighting and structural occluders (e.g., stems, trellises, or support wires), imposes high demands on vision-based perception systems. These conditions significantly degrade the accuracy and reliability of fruit detection and localization, which are critical for robotic harvesting and automated yield estimation.

Therefore, it is essential to develop occlusion-resilient perception methods tailored to greenhouse-specific scenarios. Amodal segmentation, which infers complete object masks including invisible parts, offers a promising solution to this challenge. In this study, a deep learning-based amodal segmentation method is proposed for greenhouse tomatoes, targeting the reconstruction of occluded regions to support robust visual perception and task execution in controlled-environment agriculture.

This task offers significant benefits to various downstream applications. For instance, in 3D reconstruction (Seitz et al., 2006), having complete shape information is crucial for generating more accurate 3D models, especially when objects can only be observed from limited viewpoints. In cases where the view is restricted or objects are partially occluded, understanding the full structure of the object helps enhance the model’s realism and reconstruction accuracy. For video segmentation tasks, objects in videos are often partially obscured by other elements, and having complete shape information aids in maintaining object consistency across frames, thereby improving segmentation quality and precision. In dynamic scenes, the continuity of object shapes significantly reduces errors caused by occlusion or movement. Additionally, in agricultural machine vision systems, particularly in controlled-environment agriculture, perceiving the full structure of occluded objects is essential for navigation and task execution. The complexity of greenhouse environments, where objects such as plants or machinery frequently cause occlusions, makes global shape perception vital for optimal path planning, obstacle avoidance, and harvesting strategy refinement. Accurate object perception allows the system to reliably assess fruit ripeness and determine the optimal harvesting time, thus improving harvesting efficiency, reducing manual intervention, and ultimately lowering labor costs. Traditional segmentation algorithms, such as thresholding, region growing, and edge-based methods, are primarily designed for semantic segmentation, which involves dividing images into predefined categories. While these methods can achieve reasonable results in simple scenarios, they often struggle in complex agricultural environments where multiple instances of the same class are present and occlusions are common. Some recent works have demonstrated instance-level segmentation capabilities using point cloud data (Jiang et al., 2025). Nevertheless, these methods still face challenges when dealing with heavily occluded scenes or when full object masks, including invisible regions, are required. As a result, more researchers are applying deep learning techniques to amodal segmentation tasks. Leveraging convolutional neural network (CNN) and other advanced architectures such as U-Net (Ronneberger et al., 2015) and Mask R-CNN (He et al., 2017), amodal segmentation not only performs semantic segmentation but also enables precise instance-level segmentation and even part-level segmentation within images.

The earliest work on amodal segmentation can be traced back to the research by (Li and Malik, 2016), where they synthesized images to create the first amodal instance segmentation dataset and trained and tested their proposed model, the Amodal Segmentation Network (ASN). To further validate the effectiveness of the amodal segmentation task, (Zhu et al., 2017) conducted additional studies. They invited multiple annotators to label the same image with amodal annotations, and the results showed a high level of agreement among annotators regarding regions and edges, demonstrating the task’s clear operability. They also provided amodal annotations for 5000 images from the COCO dataset, known as the COCOA dataset. Building on this, they proposed the ExpandMask network, where the input consisted of image patches and visible mask predictions, and the output was the occluded part of the target object. (Follmann et al., 2019) further improved upon Mask R-CNN, introducing a dedicated module for amodal mask segmentation called ORCNN (Occlusion Region Convolutional Neural Network). They also compiled and organized two amodal segmentation datasets, D2SA and COCOA. Subsequently, (Blok et al., 2021) and (Gené-Mola et al., 2024) applied ORCNN to broccoli and apple datasets, achieving promising results. Their experiments demonstrated that their models outperformed other methods on these datasets, further validating their effectiveness and superiority.

This deep learning-based approach to amodal segmentation has significantly improved the perception of occluded objects, offering more precise solutions for scene understanding and complex visual tasks. However, most of these studies have been tested on public datasets or applied to agricultural datasets in a very limited capacity. In the agricultural domain, amodal segmentation faces several critical limitations. For instance, the lack of large-scale, high-quality training datasets and issues of domain mismatch often result in poor Sim-to-real (Zhao et al., 2020) transfer. In real-world greenhouse environments, images typically contain numerous instances of the same class that are occluded by one another, making amodal segmentation tasks for such occluded objects far more challenging. Although existing computational models can perform well when trained on large-scale datasets under supervised learning conditions, their performance is often significantly restricted when applied to complex greenhouse scenarios, where large datasets are scarce. Particularly in unstructured agricultural environments, frequent changes in lighting conditions, the visual similarity between crops and weeds, and the unpredictability of weather add significant complexity to the model’s ability to process such scenes. Moreover, due to the diversity of crop species, the complexity of background environments, and the difficulties associated with data collection, large-scale deep learning datasets in agriculture are relatively rare. As a result, the training and evaluation of algorithms often rely on small datasets collected by researchers, which may not adequately represent the complexities of real-world situations. In dense tomato crops, for example, occlusions between similar objects are frequent, and manually annotating such complex scenes in real datasets is both costly and prone to human bias and inaccuracies. Therefore, constructing a high-quality synthetic dataset is a more suitable solution to address this problem. Synthetic data can provide precise ground-truth annotations and allow for variable control to simulate different occlusion and lighting conditions, thereby offering models more diverse and comprehensive training data.

The use of synthetic datasets effectively compensates for the challenges in real data collection in agricultural greenhouse environments and provides more consistent training and testing conditions in Sim-to-real transfer scenarios. This approach enables models to better generalize in complex greenhouse settings, improving the accuracy and robustness of amodal perception tasks. As a result, it offers more reliable technical support for automated detection, disease recognition, and fruit harvesting in agriculture. In some weakly supervised learning studies (Cinbis et al., 2016), ground-truth labels are derived from self-generated annotations. For example, in (Yang et al., 2024), self-supervised learning is used to train deep learning models for target segmentation, with self-generated labels acting as ground truth. These models are then evaluated and tested in experimental environments. However, self-generated labels are based on model predictions of object shapes, which may differ from the actual shape of the target. To bridge this gap, researchers have made various attempts. A notable effort is the tomato dataset created by (Zhou et al., 2021). They proposed a synthetic dataset method by simulating tomato growth environments using software, followed by rendering tomato images and generating segmentation labels. However, their dataset only annotated the visible parts of the instances, without addressing the occluded parts. In amodal instance segmentation, occluded regions must be annotated in alignment with the ground truth, though the ground truth itself may sometimes be inaccurate. Our new dataset offers valuable solutions for addressing the challenge of obtaining ground-truth labels for occluded tomatoes in greenhouse environments. With this dataset, models can more effectively handle occluded instances, leading to enhanced precision and robustness in machine learning models for agricultural applications.

To tackle the problem of occluded tomato segmentation in greenhouse scenarios, this study proposes a deep learning-based amodal segmentation method focused on reconstructing the occluded parts of tomatoes. Starting from the requirements of greenhouse vision systems, this research assists in detecting and locating grasp points. A synthetic dataset, Tomato-sim, was constructed in a virtual environment to meet the data needs of current vision systems. The model was then trained and tested using an RGB-D amodal instance segmentation network embedded with the CGA module. Finally, the model was validated on a tomato occlusion test dataset from real greenhouse scenes. This method aims to improve amodal segmentation performance for occluded tomatoes in complex greenhouse environments by leveraging synthetic datasets and the CGA module.

## 2 Materials and methods

### 2.1 Data acquisition

In this study, 2,000 images containing both RGB and depth information were generated across 40 different scenario conditions, using Blender Proc (Denninger et al., 2019) for photorealistic rendering. In the Blender software, various tomato models of different shapes and colors, along with their branches and leaves, were constructed. Between 1 to 10 tomatoes were randomly placed in the scene, and images were captured by randomly setting the camera pose. This approach enabled the acquisition of ground-truth RGB images for each instance or frame directly from the computer-rendered 3D scenes.

#### 2.1.1 Camera sampling and lighting condition settings

**Figure 2:**
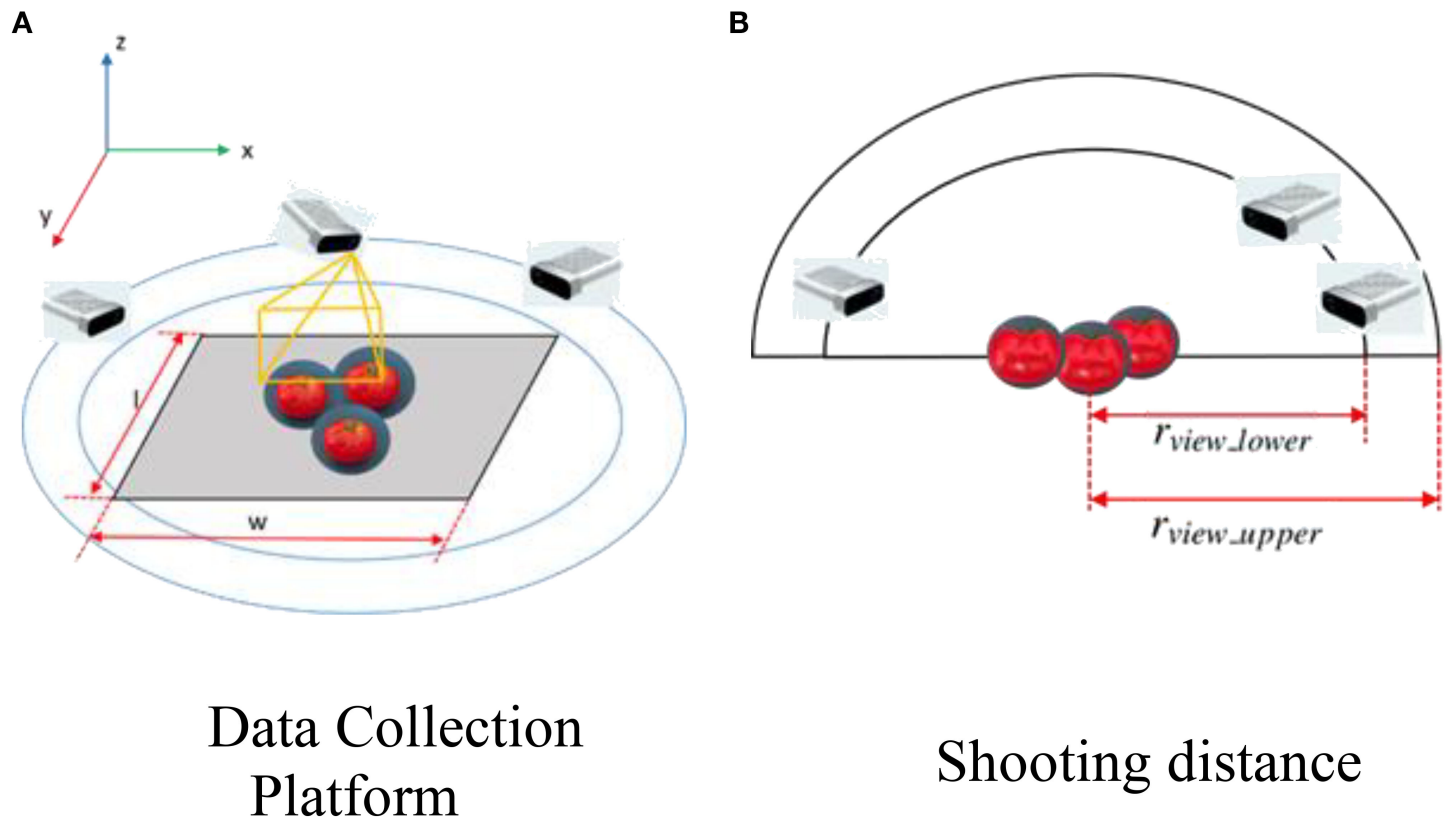
Diagram illustrating two setups. **(A)** Data collection platform with cameras positioned around tomatoes on a rectangular surface, showing dimensions width (w) and length (l) in a 3D space with axes x, y, and z. **(B)** Shooting distance with cameras viewing tomatoes at different ranges, labeled r_view_lower and r_view_upper.

To capture images of tomatoes under various occlusion conditions, we constructed synthetic greenhouse scenes using pre-designed 3D models of tomato fruits and branches. These objects were randomly placed in 3D space with varying x, y, z coordinates and orientations. Camera viewpoints were uniformly sampled from two concentric hemispheres centered on the tomato plant, ensuring sufficient angular diversity for simulating occlusions (as shown in **Figure 2**).

The sampling range was controlled by two parameters, l and w, where l∈[1,2] and w∈[2,4] meters. The inner and outer radius bounds for the viewpoint sampling were defined as shown in Equations 1 and 2.


(1) $$r_{view\_lower}=\text{max}\left(w/2,l/2\right)$$

(2) $$r_{view\_upper}=1.7\times r_{view\_lower}$$

In greenhouse environments, variations in lighting can significantly affect the visual appearance, color distribution, and surface texture of objects, which in turn influence the performance of image-based object detection and segmentation algorithms. For instance, under strong lighting conditions, the increased illumination intensity enhances contrast within the image, making object edges appear sharper and more distinct, thereby facilitating foreground-background separation and improving segmentation accuracy. In contrast, under weak lighting, the reduction in contrast leads to less pronounced object boundaries, resulting in blurred edges and a higher risk of segmentation failure or misclassification.

Compared to single-modality RGB data, the use of RGB-D inputs provides richer multi-source information. In particular, depth data remains relatively invariant to changes in lighting conditions and shadows, offering more stable structural cues for object localization and shape estimation. This property enables the model to maintain reliable performance even in complex lighting environments, where RGB images alone may suffer from intensity distortion or loss of detail due to overexposure, underexposure, or shadowing effects.

**Figure 3:**
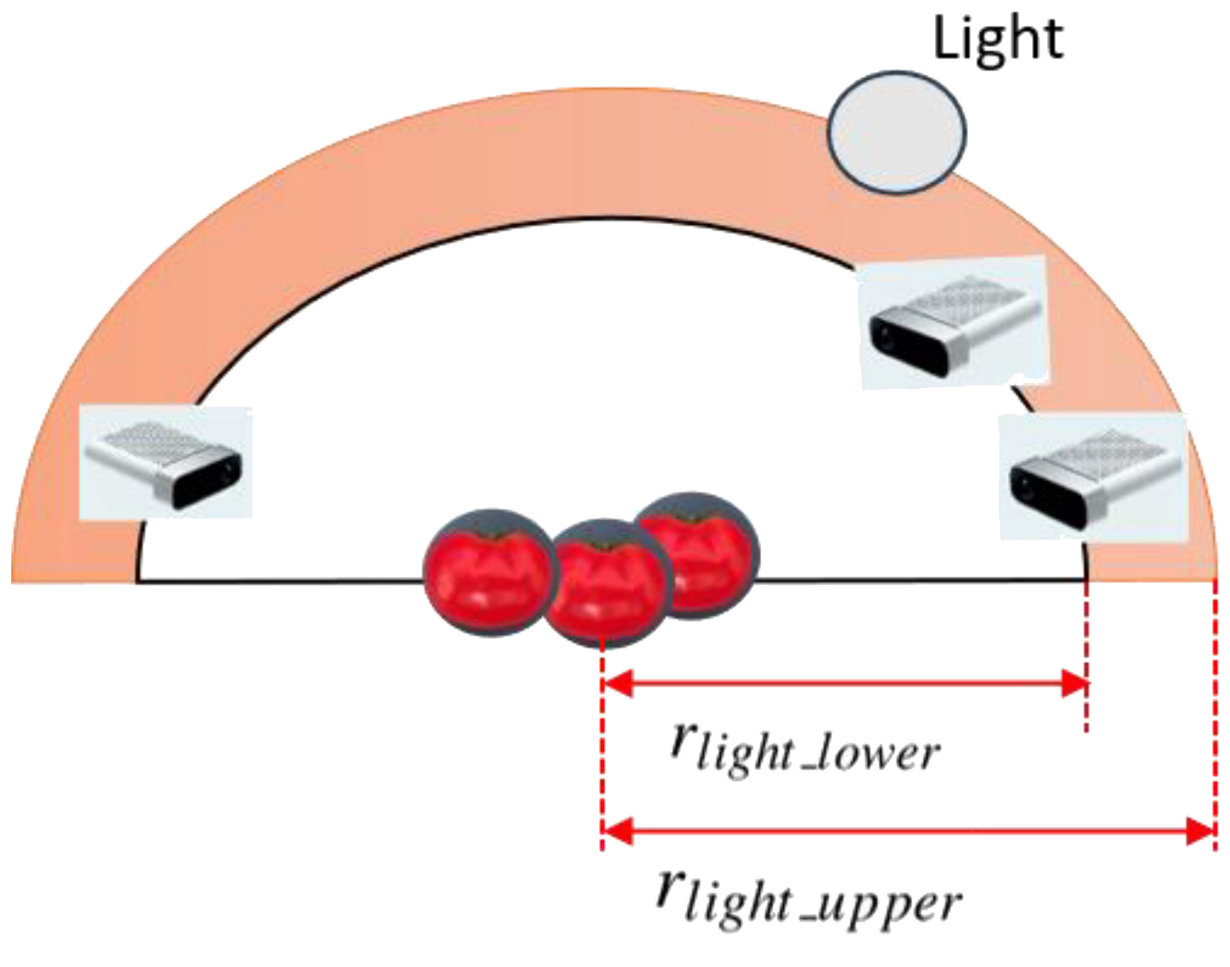
Sampling of lighting conditions.

To simulate diverse lighting conditions that realistically reflect the variability found in greenhouse settings, we introduced randomized lighting during synthetic data generation. Specifically, three distinct illumination scenarios were designed: strong, normal, and weak lighting, corresponding to different levels of intensity and contrast observed in real greenhouses. For each sampled camera viewpoint, between 0 and 2 spherical light sources were randomly added to the scene to emulate these lighting conditions (as shown in **Figure 3**, in pink). These light sources were placed using a strategy consistent with the camera viewpoint sampling, namely within the same concentric hemispherical region centered on the target object. The spatial bounds for light source placement were defined relative to the camera’s upper view radius.

The sampling constraints for the lower and upper radii of the lighting hemisphere, r_light_lower_ and r_light_upper_ are defined by Equations 3, 4:


(3) $$r_{light\_lower}=r_{light\_upper}+0.1m$$

(4) $$r_{light\_upper}=r_{light\_lower}+1m$$

**Figure 4:**
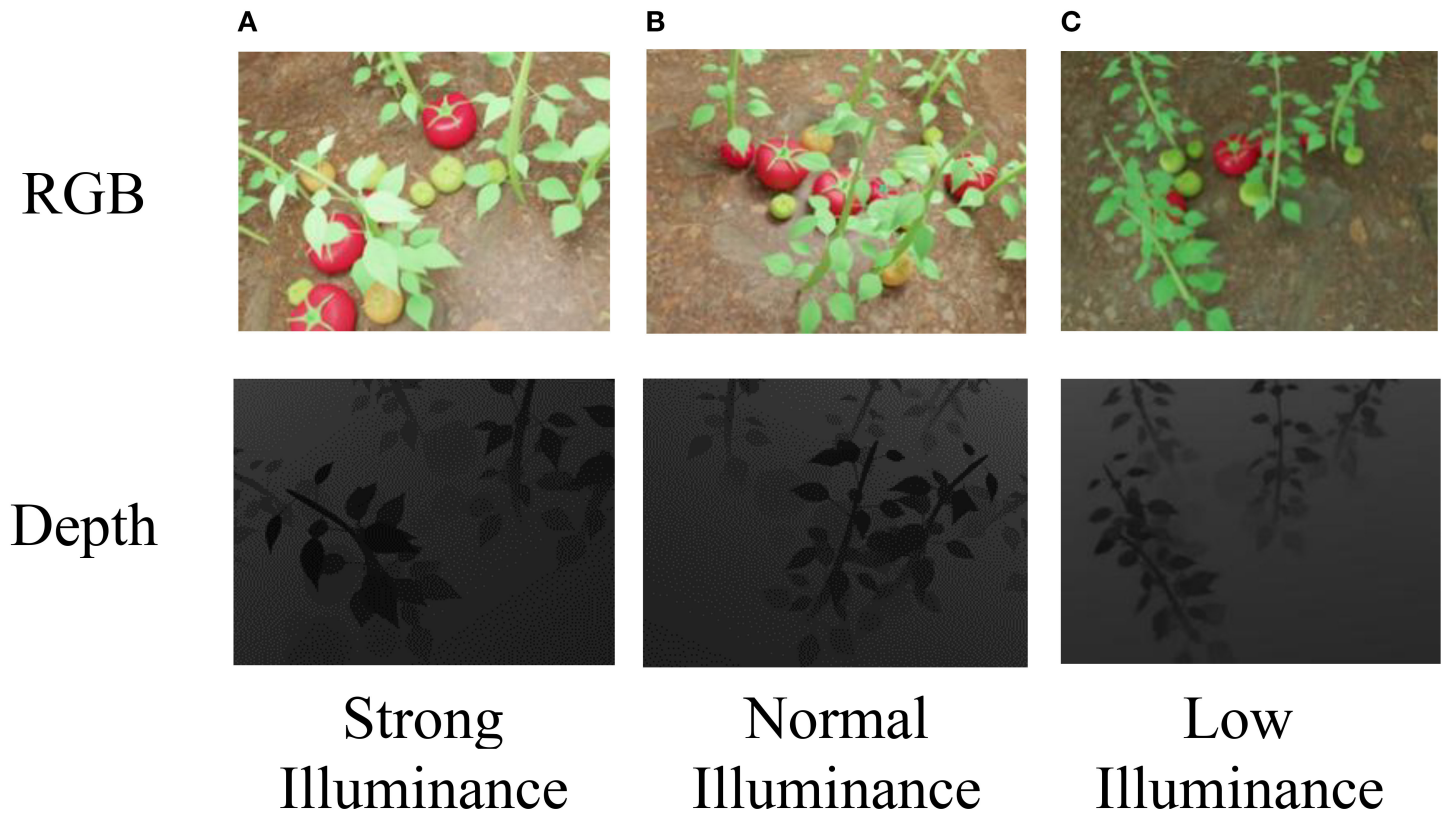
**(A)** RGB image of tomato plants under strong illuminance. **(B)** RGB image of tomato plants under normal illuminance. **(C)** RGB image of tomato plants under low illuminance. Below each panel, the corresponding depth image shows varying shading levels, with **(C)** being the darkest and **(A)** the lightest. The depth values correspond to true distances ranging from 0.25 to 5.46 m.

This setup enabled us to simulate soft shadows, directional lighting, and realistic greenhouse illumination by rendering synthetic images under three distinct lighting conditions-strong, normal, and weak-reflecting the typical variability observed in natural greenhouse environments. Each illumination condition was rendered using physically-based materials with enabled shadow casting and reflection, producing realistic phenomena such as soft shadows, directional highlights, and illumination gradients. Example outputs, including RGB and corresponding depth images under different lighting levels, are shown in **Figure 4**.

Although synthetic data cannot fully replicate real-world conditions, our dataset design incorporates variability in both lighting and viewpoints to minimize the domain gap. The model was trained solely on synthetic RGB-D images, and its generalization capability was evaluated through inference on both synthetic and real-world test sets.

In this study, we partitioned the dataset into training and testing sets at a ratio of 8:2. **Table 1** presents the distribution of RGB images in the training set, while **Table 2** shows the distribution in the testing set. Since the position of each tomato on the plant affects both light intensity and the degree of occlusion, we categorized the images into three levels based on occlusion rate: 0–10% (low occlusion), 10–30% (moderate occlusion), and 30–100% (high occlusion). Here, 0% indicates complete visibility, while 100% represents total occlusion.

**Table 1:** The distribution of RGB image data in the training set of tomato-sim.

Occlusion rate(%)	Low illuminance	Normal illuminance	Strong illuminance	Total
[0,10]	25/84	50/158	25/78	100/320
[10,30]	50/220	125/582	50/228	225/1030
[30,100]	125/694	425/2155	125704	625/3553
Total	200/998	600/2895	200/1010	1000/4903

**Table 2:** The distribution of RGB image data in the test set of tomato-sim.

Occlusion rate(%)	Low illuminance	Normal illuminance	Strong illuminance	Total
[0,10]	5/18	12/38	5/16	22/72
[10,30]	10/48	24/95	10/45	44/188
[30,100]	25/102	84/342	25/110	134/554
Total	40/168	120/475	40/171	200/814

Columns and rows contain image categories (number of images/number of instances). Each row corresponds to a different level of occlusion in the tomatoes.

These thresholds were determined based on both the natural clustering of occlusion levels observed in our manually annotated real-world greenhouse images and commonly adopted practices in agricultural vision research (e.g., Yang et al., 2024; Li et al., 2022). For consistency, the same occlusion thresholds were also applied to the synthetic dataset. The same grouping standard is used consistently in **Table 2** for data organization and performance evaluation.

#### 2.1.2 The acquisition of occlusion masks

**Figure 5:**
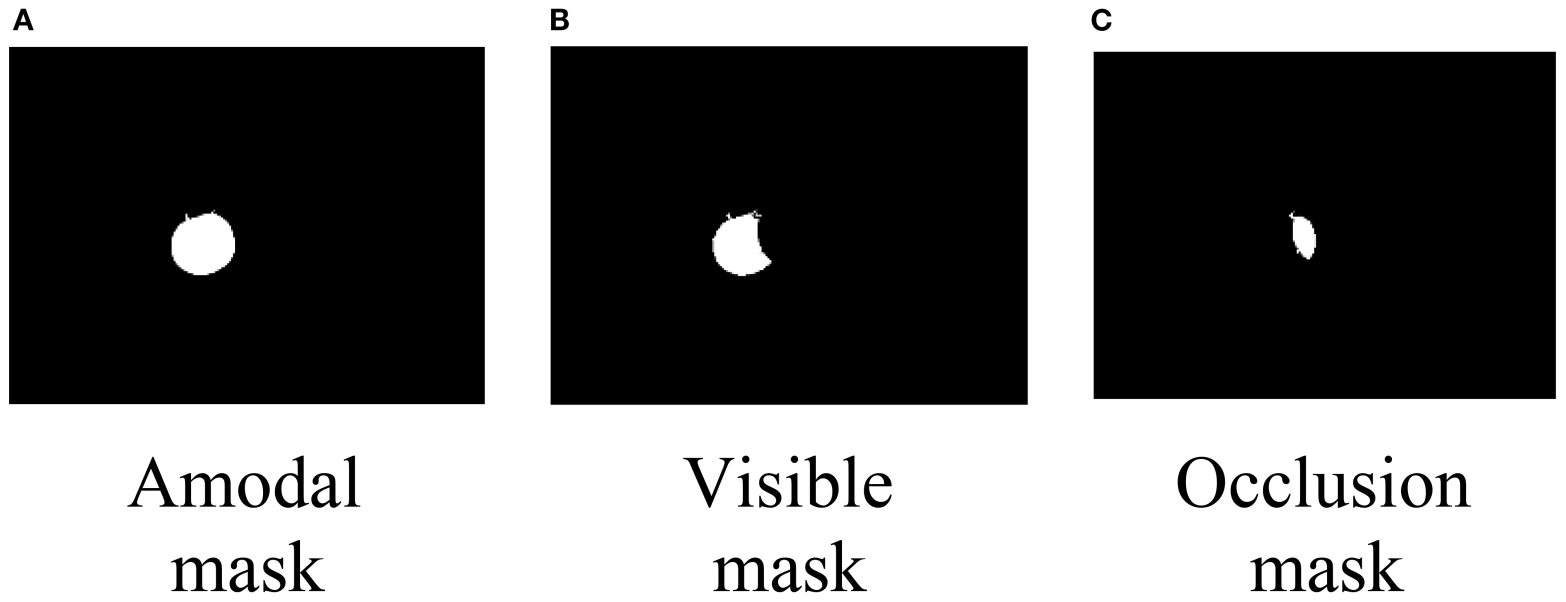
**(A)** Amodal mask shown as a full white circle on a black background. **(B)** Visible mask shown as a partial white crescent. **(C)** Occlusion mask shown as a smaller white shape.


The synthetic 3D scene-generated dataset offers a high degree of annotation flexibility, providing amodal instance masks, complete appearances, occlusion order, and layer order for all objects in the scene. For each view, the system captures RGB and depth images of the desktop scene, utilizing the built-in instance segmentation feature of NVIDIA’s Isaac Sim Replicator Composer to obtain instance segmentation masks for the entire scene. Subsequently, amodal and modal masks for each object are extracted from the instance segmentation masks. The occlusion mask and occlusion rate of each object are then calculated. The occlusion mask is obtained by subtracting the modal mask from the amodal mask, as illustrated in **Figure 5** and formulated in Equation 5.

(5) $$M_{o}=M_{A}-M_{V}$$

The occlusion rate is calculated by dividing the number of pixels in the occlusion mask by the number of pixels in the amodal mask. If an object’s occlusion rate equals 1, it means the object is completely occluded from the viewpoint, and the annotation for that object is not saved for that view. In such cases, the object’s visibility is disabled to capture the mask for the next object.

### 2.2 Greenhouse tomato dataset under real scenarios

#### 2.2.1 Collection equipment

The greenhouse tomato dataset used in this study was entirely collected using the Azure Kinect DK depth camera. During image acquisition, the Azure Kinect depth camera was utilized to capture RGB-D images, with the color camera set to a resolution of 1920×1080 pixels at 30 frames per second (fps) and the depth camera set to a resolution of 640×576 pixels at 30 fps.

Azure Kinect, developed by Microsoft, is a depth camera capable of simultaneously capturing both RGB and depth data. It features a high-resolution and high-sensitivity lens, capable of capturing high-quality depth information within a range of 0 to 10 meters. The depth camera of Azure Kinect uses time-of-flight (ToF) technology, which projects modulated light in the near-infrared spectrum onto the scene and records the time it takes for the light to travel from the camera to the scene and back. This travel time, along with the speed of light, is used to calculate depth values for different positions in the scene, generating a depth map. To ensure the generalizability of the data, the tomato plants were randomly photographed from multiple angles and positions under different lighting conditions within the greenhouse. Each image set includes RGB and corresponding depth images. The captured RGB and depth images were registered, ensuring that the pixels in the RGB image corresponded to the distance-representing pixels in the depth image. Finally, the images were cropped to 640×480 pixels for both RGB and depth.

#### 2.2.2 Greenhouse data acquisition

**Table 3** presents the data distribution of the real-world test set, where the intensity of light and the level of occlusion vary across different positions on the tomato plants. Based on the degree of occlusion, the images are categorized into three levels: 0-10%, 10-30%, and 30-100%, with 0% indicating no occlusion and 100% indicating full occlusion.

**Table 3:** Construction of tomato datasets under real greenhouse scenarios.

Occlusion rate(%)	Low illuminance	Normal illuminance	Strong illuminance	Total
[0,10]	5/10	12/25	5/11	22/46
[10,30]	8/18	24/65	8/16	40/99
[30,100]	27/72	84/272	27/91	138/435
Total	40/100	120/362	40/118	200/580

**Figure 6:**
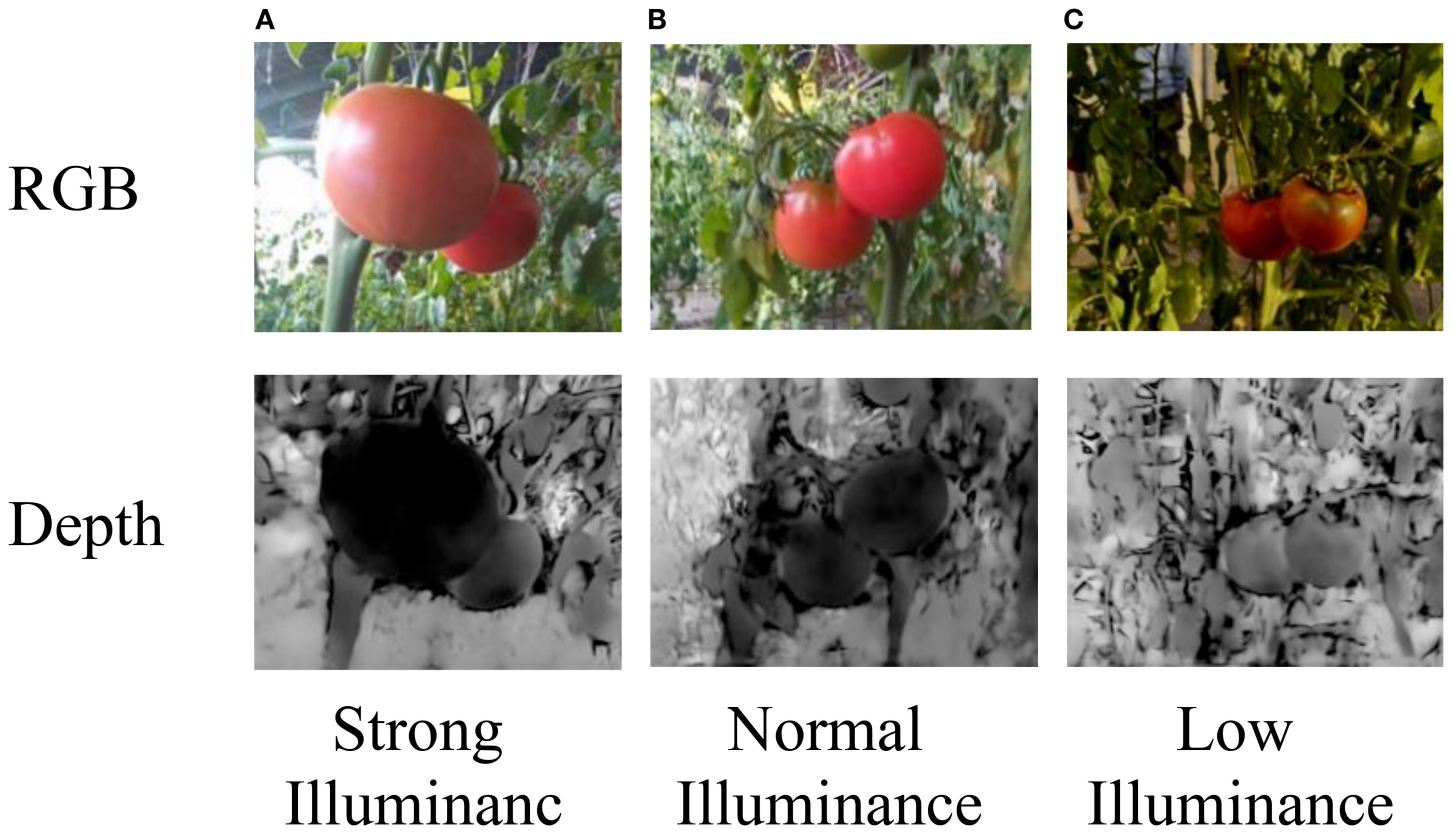
**(A)** Brightly lit tomato in RGB image. **(B)** Normally lit tomatoes in RGB image. **(C)** Dimly lit tomatoes in RGB image. The bottom row shows the corresponding depth images under strong, normal, and low illuminance. Labels indicate lighting conditions. The depth values correspond to true distances ranging from 0.25 to 5.46 m.

Tomato images captured under different lighting conditions in real greenhouse scenarios, and their corresponding depth images are also collected, as shown in **Figure 6**.

#### 2.2.3 Data annotation

**Figure 7:**
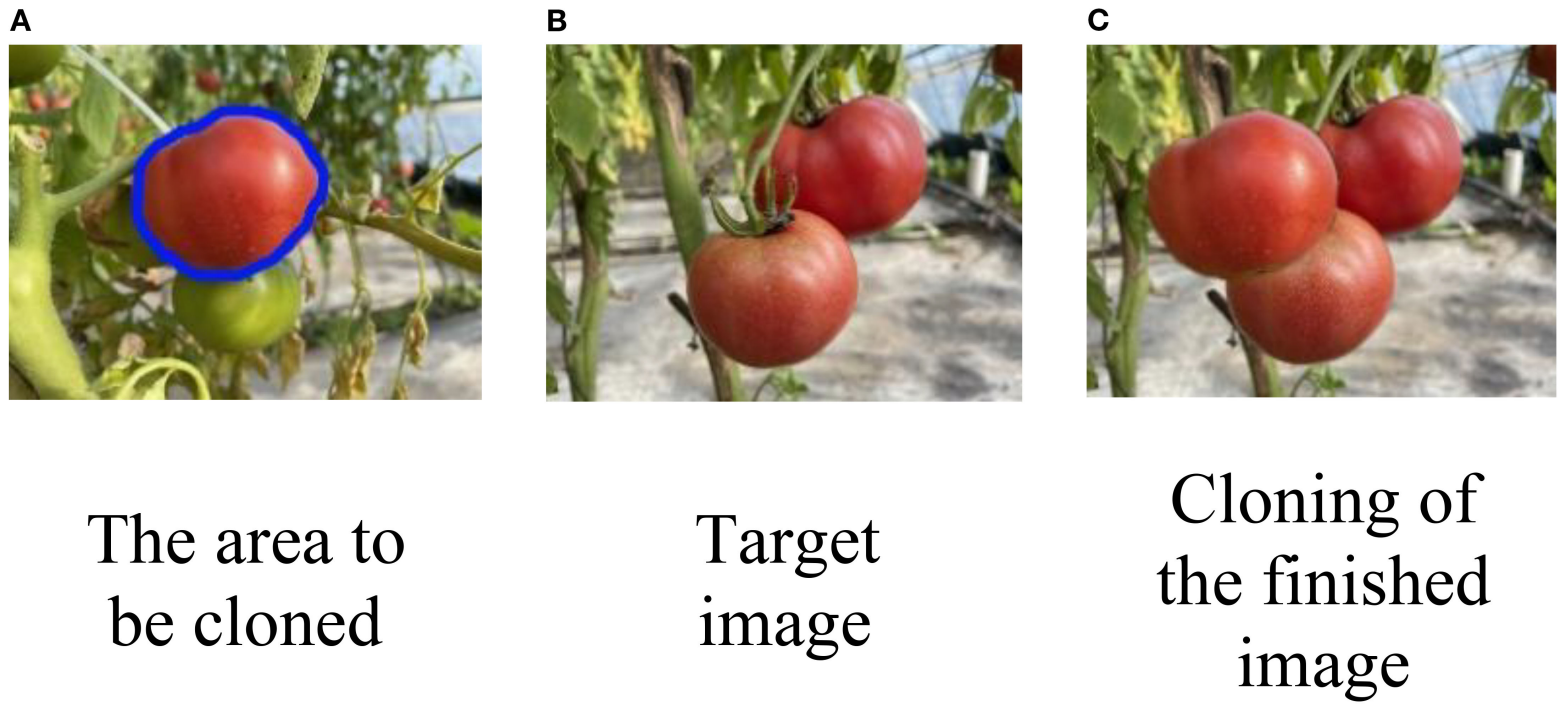
**(A)** Ripe red tomato outlined in blue for cropping, with a smaller green tomato nearby. **(B)** Target image with two ripe red tomatoes on a vine. **(C)** Final result, where the tomato from **(A)** has been cropped and pasted onto **(B)** to create an occlusion effect.

Unlike other image segmentation tasks, instance segmentation requires pixel-level masks for visible objects, while amodal segmentation not only needs visible object masks but also integrates semantic labels for both visible and occluded parts of the scene. After mean cloning and fusion (Farbman et al., 2009), the dataset easily captures more semantic information about the target images. To segment the occluded areas, the combined mask of visible and invisible regions after image fusion is subtracted from the visible mask before fusion, as illustrated in **Figure 7**.

In this study, the LabelMe tool (Russell et al., 2008) was used to annotate each region hierarchically, and 200 images with ground-truth amodal masks were selected as the test set. Annotating an entire image takes approximately 5 minutes, with each instance requiring around 0.5 minutes on average. Compared to the efficient construction of synthetic datasets, manual annotation in real-world scenes is time-consuming, highlighting the advantage of synthetic datasets in improving data annotation efficiency.

### 2.3 RGB-D-based amodal instance segmentation method for tomatoes

#### 2.3.1 RGB-D-based greenhouse tomato amodal segmentation model

**Figure 8:**
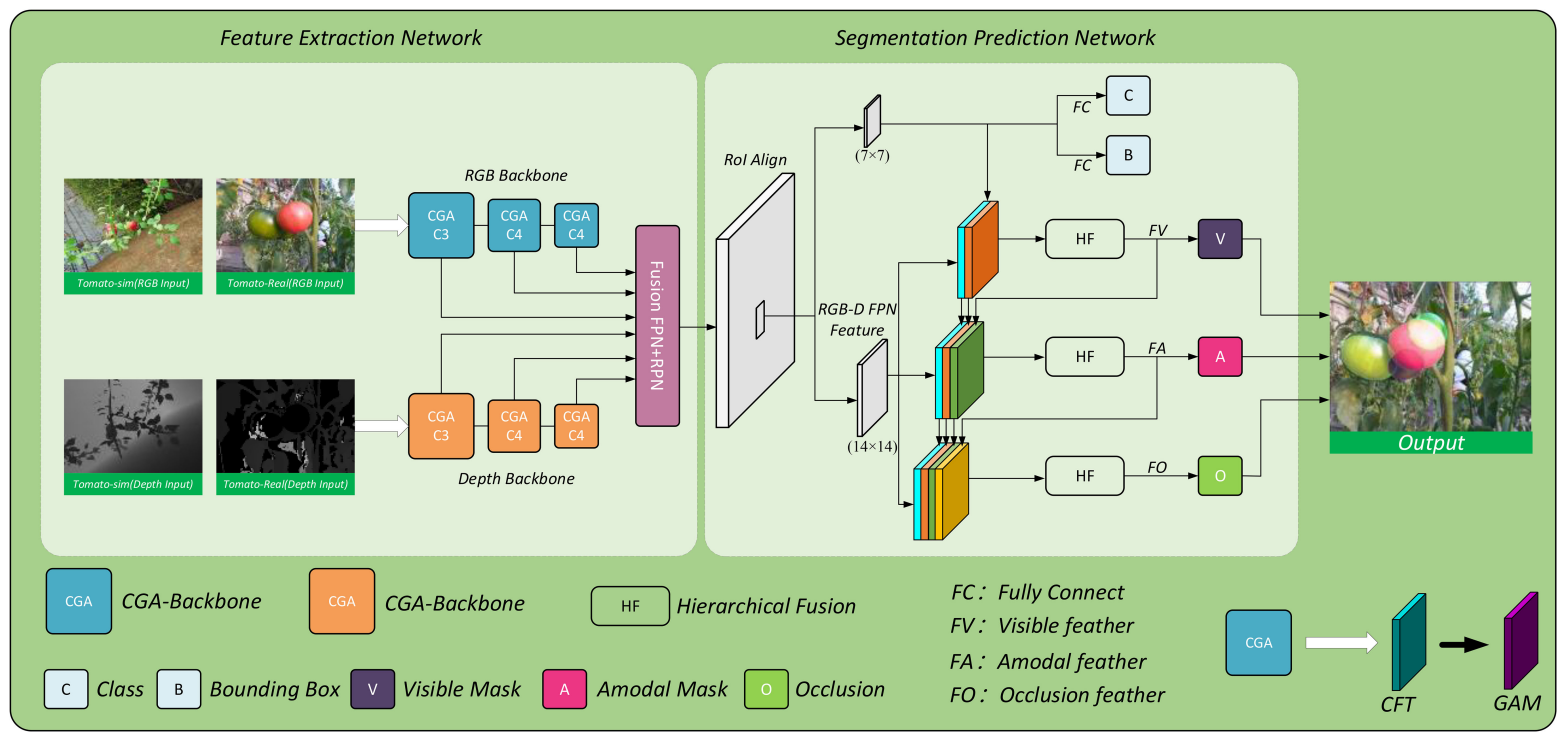
Overall architecture diagram of the CGA-ASNet model.


The CGA-ASNet architecture, as illustrated in **Figure 8**, consists of two main components: the feature extraction network and the segmentation prediction network. The feature extraction and fusion network first extracts RGB features and depth features separately from the input RGB and Depth images. The depth features from the C3, C4, and C5 layers of the CGA-50 Backbone are concatenated with the corresponding RGB features from the same layers. A 1×1 convolution is applied to fuse the RGB-D features, reducing the channel dimensions. This fusion forms an RGB-D feature pyramid, which is then passed through the Region Proposal Network (RPN) and RoIAlign (Region of Interest Align) layers to generate the RGB-D features. These multi-dimensional feature maps are then fed into the segmentation prediction network for segmentation tasks. The model incorporates the CGA module, based on the Unseen Object Amodal Instance Segmentation (UOAIS) architecture. The CGA module is composed of the CFT module (proposed in this study) and the GAM module (Liu Y. et al., 2021). The improved model is highly adaptable to the constructed synthetic dataset, ensuring both high accuracy and enhanced training and inference speed, even with smaller datasets. The model effectively handles variations in lighting conditions and tomato color changes in greenhouse environments. Additionally, the introduction of a shape convolution module strengthens the model’s perception of tomato shape and position, reducing the impact of occlusions caused by branches and leaves. The model’s loss function is defined as shown in Equation 6.

(6) $${\text{L}}_{\text{loss}}={\text{L}}_{\text{cls}}+{\text{L}}_{\text{box}}+{\text{L}}_{\text{V}}+{\text{L}}_{\text{A}}+{\text{L}}_{{\text{O}}_{\text{cls}}}+{\text{L}}_{{\text{rpn}}_{\text{cls}}}+\text{L}\_\left(\text{rpn}\_\text{loc}\right)$$

Among them, the abbreviation L_cls_ refers to the loss of classes, L_box_ refers to the loss of bounding boxes, L_V_ refers to the loss of non-modal mask losses, and L_Ocls_ refers to the loss of occlusion classification.

#### 2.3.2 CFT attention

**Figure 9:**
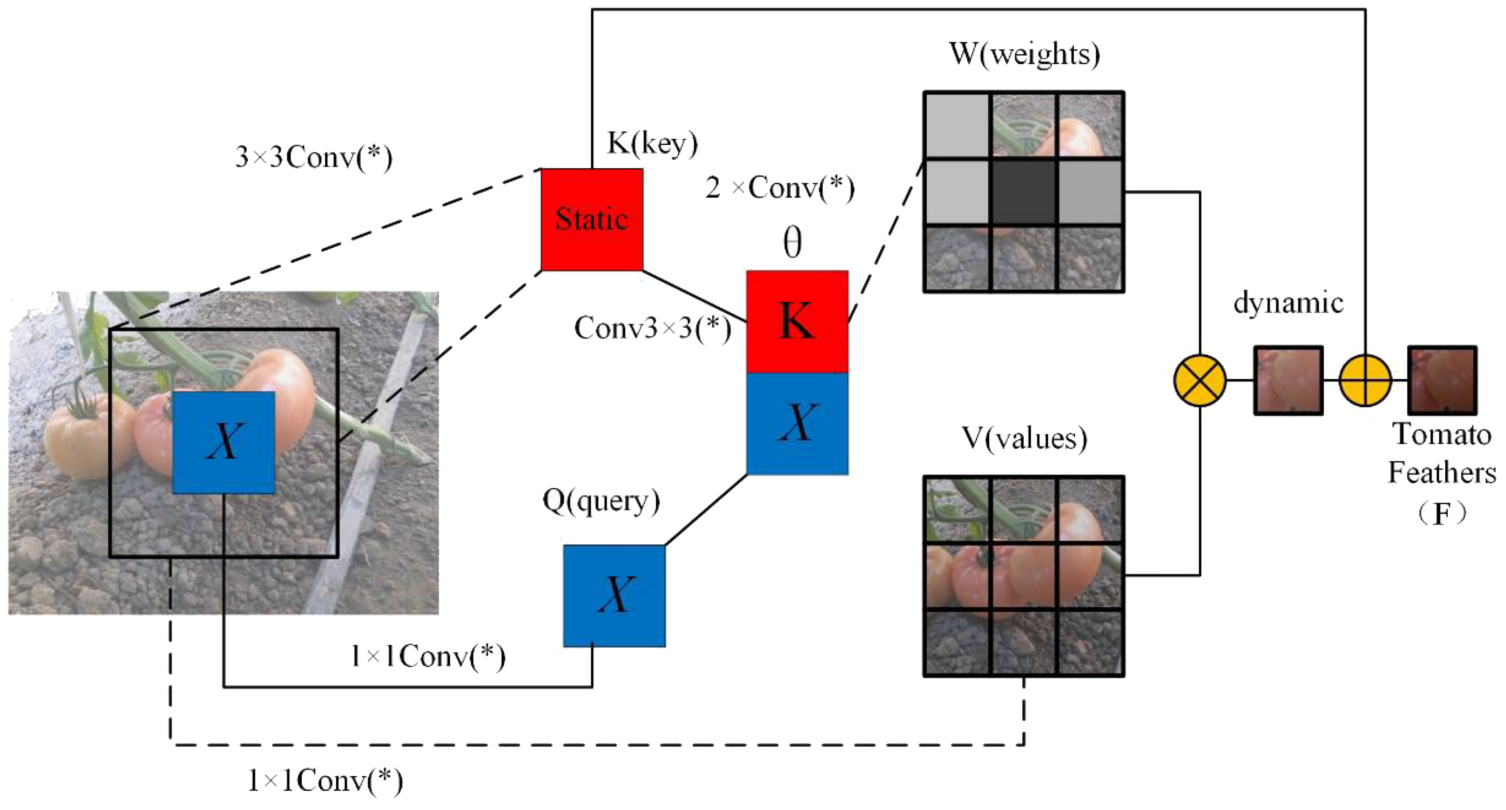
Structure of CFT self-attention module. Here, the * is not alone. When combined with conv, it forms conv(*), representing a 1x1 convolution.

To enhance the global modeling capability of ResNet50, we propose a Contextual Features Transformer (CFT) module, the structure of which is illustrated in **Figure 9**. This module replaces the standard 3×3 convolution in the residual block. Unlike conventional self-attention mechanisms that compute attention weights based on dot-product similarity, the proposed CFT module leverages learnable convolutions to generate attention scores. This design integrates the inductive bias of convolution with the long-range dependency modeling strength of attention, effectively avoiding the scale sensitivity issues of dot-product attention while providing more stable and spatially aware representations. Moreover, it introduces only minimal computational overhead, making it particularly suitable for dense prediction tasks.

The proposed Convolutional Feature Transformer (CFT) module is designed to simultaneously model local details and global dependencies by leveraging a convolution-based attention mechanism in place of traditional dot-product attention. Specifically, the input feature map *X* is first passed through a 3×3 convolution to extract the key feature *K*, preserving spatial context. A second 3×3 convolution is applied to further enhance local information within the key representation. In parallel, the query *Q* and value*V* features are obtained from *X* using two separate 1×1 convolutions for dimensionality reduction while preserving feature structure.

After computing these features, spatial dependencies are modeled by concatenating the key and query features along the channel dimension. This combined representation is passed through two 1×1 convolutions to produce the spatial interaction logits θ, which are normalized by a Softmax function to yield the attention weight matrix *W*. The matrix *W* is then applied to the value feature *V* via weighted summation. To further incorporate global context, the value feature is enhanced using a dilated convolution layer *M*, which increases the receptive field without reducing resolution. The result is fused with the original key *K* using a final 1×1 convolution, producing the final output *Y*, denoted as the tomato feature *F* for subsequent segmentation tasks.

The core idea of CFT is to replace the conventional dot-product attention with convolution-based attention, allowing the network to better integrate spatial inductive bias and global dependencies. The complete formulation is given as shown in Equations 7–9.


(7) θ=Conv(Conv(K⊕Q))

(8) W=Softmax(θ)

(9) Y=Conv(K+M⊗V)

where Conv(*) denotes a 1×1 convolution; ⊕ represents concatenation; ⊗ denotes matrix multiplication.

#### 2.3.3 GAM attention

**Figure 10:**
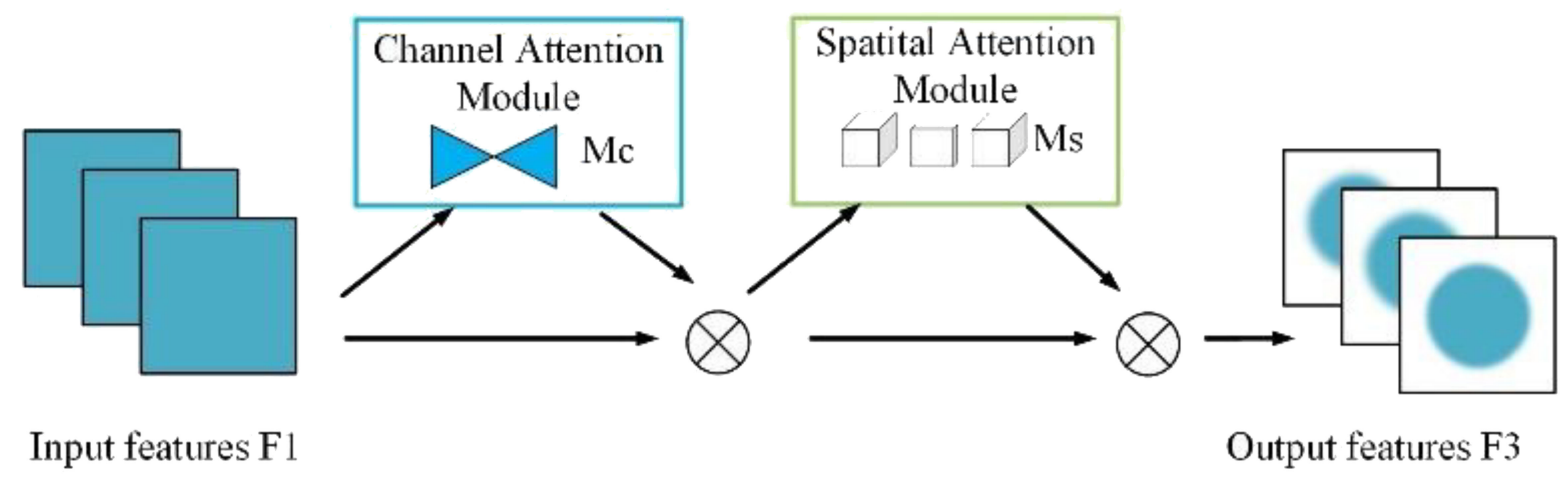
Global attention mechanism.

The Global Attention Mechanism (GAM) enhances feature representations by applying attention along both channel and spatial dimensions. It consists of two independent submodules: the Channel Attention Module (CAM) and the Spatial Attention Module (SAM). The overall architecture is illustrated in **Figure 10**.

**Figure 11:**
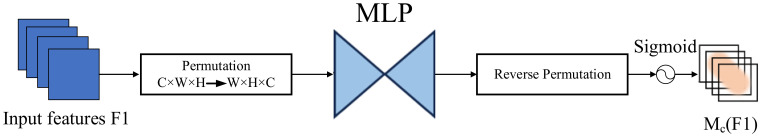
Channel attention module.

As illustrated in **Figure 11**, CAM first applies global average pooling and global max pooling across spatial dimensions of the input feature map $\text{F}\in {\text{R}}^{\text{C×H×W}}$ , resulting in two descriptors of size R^C^. These descriptors are then passed through a shared two-layer MLP, where the first layer reduces the dimension by a ratio r, and the second layer restores it to C. After element-wise summation and a sigmoid activation, the resulting attention map M_c_ is used to reweight the input feature map channel-wise.

**Figure 12:**
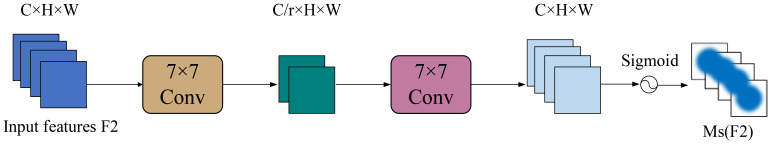
Spatial attention module.

SAM further refines the output from CAM by emphasizing important spatial locations. As shown in **Figure 12**, it applies average pooling and max pooling across channels, producing two $R^{H\times W}$ feature maps, which are concatenated and passed through a 7×7 convolution followed by a sigmoid activation to generate the spatial attention map M_s_. This map is multiplied element-wise with the input to produce the final attention-weighted output.

#### 2.3.4 Segmentation prediction network

The segmentation prediction network is composed of four main branches: the Bounding Box Prediction Branch, the Visible Mask Prediction Branch, the Amodal Mask Prediction Branch, and the Occlusion Classification Prediction Branch. The Bounding Box Prediction Branch takes the 7×7 feature map output from the RPN (Region Proposal Network) and passes it through two fully connected layers to predict the bounding box B and class C. The feature map is then upsampled to a 14×14 feature map to provide bounding box features for the subsequent branches, ensuring that instance masks are segmented within the predicted bounding box. The Visible Mask Prediction Branch, the Amodal Mask Prediction Branch, and the Occlusion Classification Prediction Branch utilize the 14×14 feature map from the RPN, along with features fused from the previous branches, to predict the visible mask V, the amodal mask A, and the occlusion classification O, respectively. The mathematical formulations for each branch are expressed in Equations 10–13.


(10) $${\text{F}}_{\text{V}}=\left({\text{h}}_{\text{V}}\left({\text{F}}_{\text{B}},{\text{F}}_{\text{RoI}}\right)\right)$$

(11) $${\text{F}}_{\text{A}}=\left({\text{h}}_{\text{A}}\left({\text{F}}_{\text{B}},{\text{F}}_{\text{RoI}},{\text{F}}_{\text{V}}\right)\right)$$

(12) $${\text{F}}_{\text{O}}=\left({\text{h}}_{\text{O}}\left({\text{F}}_{\text{B}},{\text{F}}_{\text{RoI}},{\text{F}}_{\text{V}},{\text{F}}_{\text{A}}\right)\right)$$

(13) $$\text{V},\text{A},\text{O}={\text{P}}_{\text{V}}\left({\text{F}}_{\text{V}}\right),{\text{P}}_{\text{A}}\left({\text{F}}_{\text{A}}\right),{\text{P}}_{\text{O}}\left({\text{F}}_{\text{O}}\right)$$

In the segmentation prediction network, F_B_, F_RoI_, F_V_, F_A_, and F_O_ represent the bounding box feature, the RoI feature, the visible mask feature, the amodal mask feature, and the occlusion mask feature, respectively. The hierarchical fusion modules hv, h_A_ and ho correspond to the visible mask, amodal mask, and occlusion classification branches. Specifically, the hierarchical fusion module integrates each input feature and reduces the channel dimensions through three 3×3 convolution layers to decrease the parameter count. These are then fed into another set of three 3×3 convolution layers to generate the task-specific features for each branch. The prediction layers P_V_, P_A_ and P_o_ are responsible for predicting the visible mask, amodal mask, and occlusion classification, respectively. P_V_ and P_A_ use 2×2 deconvolutions and a fully connected layer, while P_o_ consists of a fully connected layer to output the final results.

## 3 Results and analysis

### 3.1 Training and parameter setting

This study aims to address the lack of real RGB-D datasets by applying deep learning models, specifically focusing on tomatoes. Through software, synthetic RGB-D images simulating occluded tomatoes in a greenhouse environment are generated to build a diverse and high-quality dataset. The convolutional neural network (CNN) extracts and integrates features from both RGB and depth images using feature extraction algorithms. Multiple detection branches are employed to predict the visible part masks and the contours of the occluded parts of the objects. A hierarchical occlusion modeling mechanism is applied to improve the accuracy of amodal segmentation for tomatoes.

During model training, comparisons between different datasets (synthetic and real-world datasets) are conducted for both training and testing. To ensure fairness in the training and testing process, all tasks are performed on the same hardware platform. The experimental platform consists of a Dell Precision 7920 with 64GB RAM, a 2.1GHz CPU with 16 cores, and an NVIDIA A6000 GPU with 48GB GDDR6 VRAM and 10,752 CUDA cores. Initial training parameters are listed in **Table 4**.

**Table 4:** Training process related parameters.

Parameter name	Parameter values
Image Size	640×480
Batch Size of Images	2
Initial Learning Rate	0.00125
Maximum Number of Iterations	90000

### 3.2 Evaluation metrics for tomato amodal segmentation quality

We adopt several evaluation metrics to quantitatively assess the instance-level segmentation performance, including Precision, Recall, F1-Score, F@75 (Ochs et al., 2013), and mean Intersection over Union (mIoU), as defined in Equations 14–18. Precision measures the proportion of correctly predicted positive instances among all predicted positives, while Recall measures the proportion of correctly predicted positive instances among all actual positives. The F1-Score is the harmonic mean of Precision and Recall, providing a balanced evaluation of model performance.

F@.75 is an instance-level metric based on the F1-score, representing the proportion of ground-truth instances that are successfully matched with predicted instances having an F1-score no less than 0.75. The pairwise F1-scores between predicted and ground-truth instances are computed, and the optimal one-to-one assignment is determined using the Hungarian algorithm. Finally, mean Intersection over Union (mIoU) is used to evaluate segmentation quality across all classes.


(14) $$\text{Precision}=\frac{\text{TP}}{\text{TP}+\text{FP}}$$

(15) $$\text{Recall}=\frac{\text{TP}}{\text{TP}+\text{FN}}$$

(16) $$\text{F}1-\text{Score}=\frac{2\text{Precision}\times \text{Recall}}{\text{Precision}+\text{Recall}}$$

(17) $$\text{F}@.75=\frac{\sum_{\left(\text{i},\text{j}\right)\in \text{M}}1\left\{{\text{F}}_{\text{i},\text{j}}\geq 0.75\right\}}{\text{N}}$$

(18) $$\text{mIoU=}\frac{1}{k+1}\;\sum_{\text{i}=0}^{\text{k}}\frac{\text{TP}}{\text{FN+FP+TP}}$$

where TP is the model correctly predicts positive instances; FP is model incorrectly predicts positive instances; FN is the model incorrectly predicts negative instances; FP is the model correctly predicts negative instances. F_i,j_ is the F1-score between predicted instance i and ground-truth instance j, M is the optimal one-to-one matching obtained via the Hungarian algorithm, and N is the total number of ground-truth instances.

In order to compare our method with other existing methods, we adopted the AP (Average Precision) and mAP (mean Average Precision) as evaluation metrics, which are commonly used for amodal segmentation tasks (Ke et al., 2021), as defined in Equations 19, 20.


(19) $$\text{AP}=\int_{0}^{1}\text{P}\left(\text{r}\right)\text{dr}$$

(20) $$\text{mAP}=\frac{1}{\text{k}}\sum_{\text{i}=1}^{\text{k}}{\text{AP}}_{\text{i}}$$

### 3.3 Analysis of test results with different backbone networks

To investigate the impact of different feature extraction backbone networks on the performance of the CGA-ASNet model, we conducted a series of controlled experiments using six different backbone architectures: ResNet50, ResNet101 (He et al., 2016), ResNeXt50, ResNeXt101 (Xie et al., 2017), ConvNeXt-Tiny (Liu et al., 2022), and Swin-Tiny (Liu Z. et al., 2021). All experiments were performed under identical training and testing conditions, with RGB-D as the input modality and only the backbone network varied.

As shown in **Table 5**, ResNet50 consistently outperformed the other backbone networks in both amodal mask prediction and occlusion segmentation. Specifically, it achieved the highest amodal F@.75 score of 92.0 and a mean Intersection-over-Union (mIoU) of 81.4%. Although newer backbone architectures such as ConvNeXt-Tiny and Swin-Tiny showed competitive results, they did not surpass the performance of ResNet50 in our task setting. This suggests that ResNet50 remains a strong and stable backbone choice for occlusion-aware segmentation tasks, particularly in our CGA-ASNet framework.

**Table 5:** Comparison results of different backbone networks.

Backbone	Amodal	
	F@.75	mIoU(%)
ResNet101	81.9	76.7
ResNext50	87.0	75.7
ResNext101	87.7	79.1
ConvNext_Tiny	88.4	78.9
Swin_Tiny	89.3	79.4
ResNet50	**92.0**	**81.4**

The bolded part is the most effective part in the backbone network and thus is supported.

### 3.4 Ablation study

Ablation experiments, commonly used to assess the influence of different components in a model, are an effective method for exploring the contributions of each module and gaining a deeper understanding of the model’s behavior. As such, ablation experiments play a crucial role in the design of neural network structures. To verify the effectiveness of the CGA module, this study designed a series of ablation experiments. We used ResNet50 as the backbone network with R-50.pkl serving as the initial weight baseline. The experiments were divided into three parts: first, the CFT self-attention module and GAM attention module were individually embedded for testing; finally, both CFT and GAM were combined and embedded into the network for comparison to evaluate their specific contributions to improving network performance.

As shown in **Table 6**, the first row presents results from the baseline model without any modifications, achieving an F@.75 score of 92.0 and a mIoU of 81.4% for amodal masks. In the second experiment, where the CFT module was added, the F@.75 score increased to 93.5 and the mIoU to 82.6%, representing improvements of 1.5 and 1.2%, respectively. The third experiment introduced the GAM module, which raised the F@.75 score to 94.2, an increase of 2.2, and the mIoU to 82.4%, a 1% improvement. Finally, the model with the combined CFT and GAM modules, forming the CGA module, achieved an F@.75 score of 94.2 and a mIoU of 82.4%. These results demonstrate that the CGA module effectively captures more semantic information from tomatoes, significantly enhancing the segmentation performance.

**Table 6:** Ablation study.

Method	Amodal	
	F@.75	mIoU(%)
Baseline	92.0	81.4
Baseline+cft	93.5	82.6
Baseline+gam	94.2	82.4
Baseline+CGA	94.2	83.3

### 3.5 Amodal segmentation results on test images with different degrees of occlusion

To evaluate the robustness of the improved amodal segmentation network, this study compared the baseline model with the CGA-embedded segmentation model across three subsets with occlusion levels greater than 0-10%, 10-30%, and 30-100%, using identical parameters. The results are shown in **Table 7** and **Table 8**. When the occlusion rate was below 10%, CGA-ASNet achieved an F@.75 score of 98.4 and a mIoU of 86.8%, both higher than the baseline model. For occlusion levels between 10% and 30%, and those above 30%, CGA-ASNet also outperformed the baseline model by 1.4 and 2.6, respectively.

**Table 7:** Baseline prediction results.

Occlusion Rate(%)	Amodal	
	F@.75	mIoU(%)
[0,10]	98.2	85.3
[10,30]	93.1	82.8
[30,100]	86.7	78.1

**Table 8:** CGA-ASNet prediction results.

Occlusion rate(%)	Amodal	
	F@.75	mIoU(%)
[0,10]	98.4	86.8
[10,30]	94.5	83.4
[30,100]	89.3	79.8

**Figure 13:**
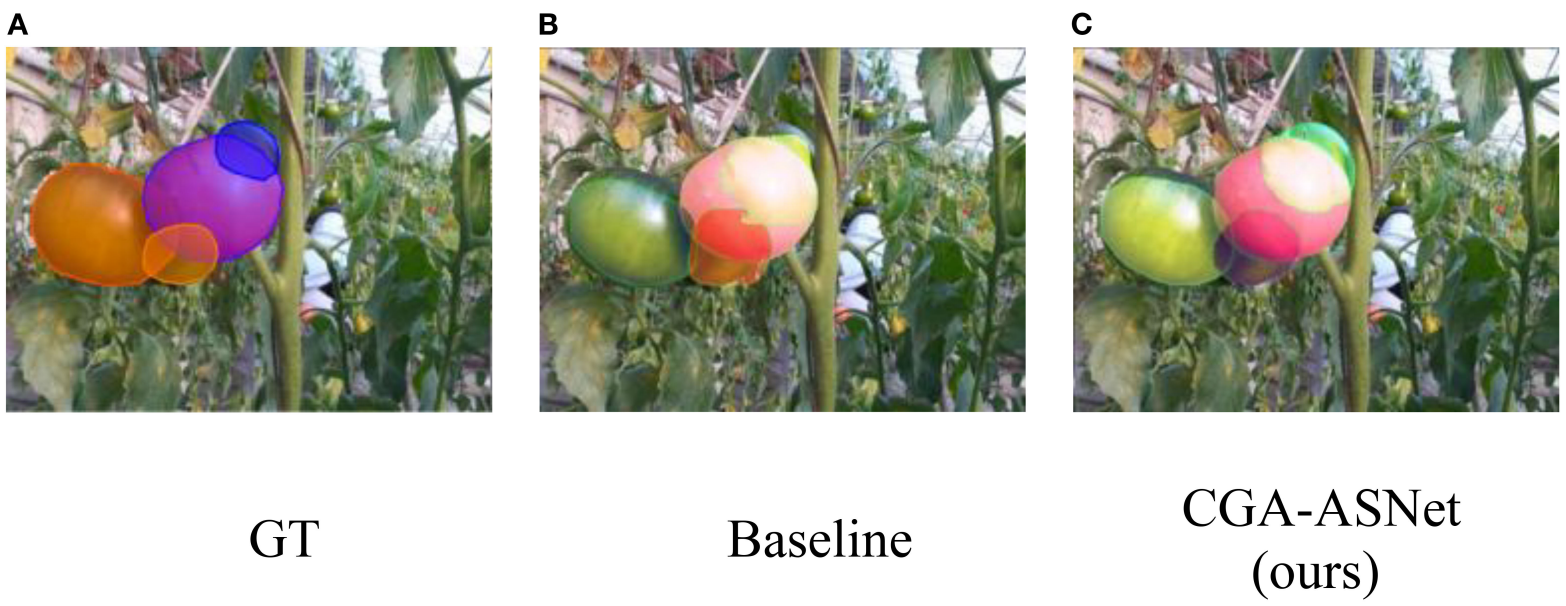
**(A)** Tomatoes with GT overlays in blue and orange. **(B)** Baseline result with mainly orange and green overlays. **(C)** CGA-ASNet result with accurate red and green overlays, showing segmentation improvements.

The results indicate that, while segmentation performance declines as occlusion increases, CGA-ASNet consistently handles severe occlusions better than the baseline. As shown in **Figure 13**, when multiple tomatoes are stacked, the baseline model without the CGA module exhibited jagged contours in its predictions of occluded tomatoes, whereas our model generated smoother and more natural predictions. This demonstrates that the CGA module significantly enhances the model’s ability to perceive and predict the edge shapes of segmented objects, improving overall prediction accuracy.

### 3.6 Comparison of test results from different models

During the experimental design phase, we reviewed several recent representative amodal segmentation models, including pix2gestalt (Ozguroglu et al., 2024), AISDiff (Tran et al., 2024), and BLADE (Liu et al., 2024), etc. However, most models only support feature extraction of the RGB channels. These models cannot provide the feature support for image segmentation based on depth information. If the RGBD four-channel data is compressed into three channels for feature extraction, the obtained features cannot accurately represent the pixel semantics of the original image. To ensure reproducibility and fair comparison, we selected a group of well-established and publicly available models as baselines for evaluation.

In this study, the Tomato-sim dataset was trained on state-of-the-art (SOTA) models, including BC-net, AISFormer (Tran et al., 2022), ORCNN (Gené-Mola et al., 2023), and Uoais-net (Back et al., 2022), using identical parameters to compare different training data (Tomato-sim and real datasets). The models were tested on datasets constructed through mean clone fusion in both synthetic and real greenhouse scenarios. **Table 9** presents the prediction results of different models.

**Table 9:** Comparison of predictions from different models.

Method	Eval	AP50(%)	AP75(%)	mAP(%)
BC-net	Tomato-sim	87.4	78.7	70.2
	real	85.7	75.5	66.7
AISFormer	Tomato-sim	92.3	86.1	74.4
	real	89.9	85.7	72.5
ORCNN	Tomato-sim	73.3	63.4	55.7
	real	72.3	58.3	52.4
Uoais-net	Tomato-sim	92.9	82.3	74.5
	real	89.6	78.3	73.1
CGA-ASNet	Tomato-sim	94.3	83.6	78.3
	real	93.1	78.4	75.0

**Figure 14:**
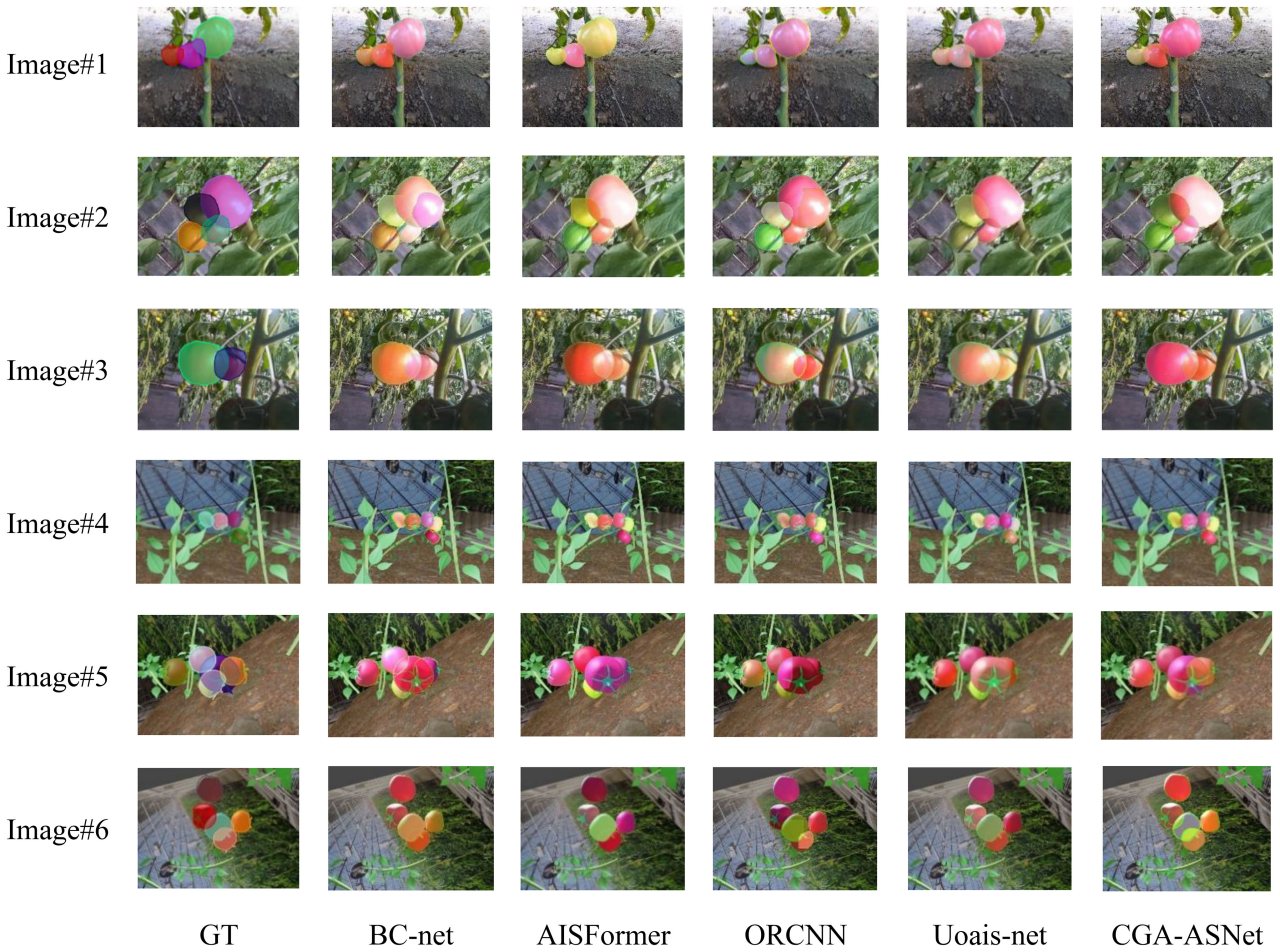
Amodal segmentation results of different models.

**Figure 14** shows the performance of these segmentation models in the amodal segmentation task. From image (2), it can be observed that in the complex stacking scenario of tomatoes, our model exhibited strong robustness. Images (1) to (3) show prediction results from real greenhouse environments, while Images (4) to (6) display performance in virtual scenes. Although all models performed well in the virtual scenario, our model demonstrated the best segmentation ability, especially in handling complex occlusion and multi-layer stacking, achieving significantly higher segmentation accuracy compared to other models.

**Figure 15:**
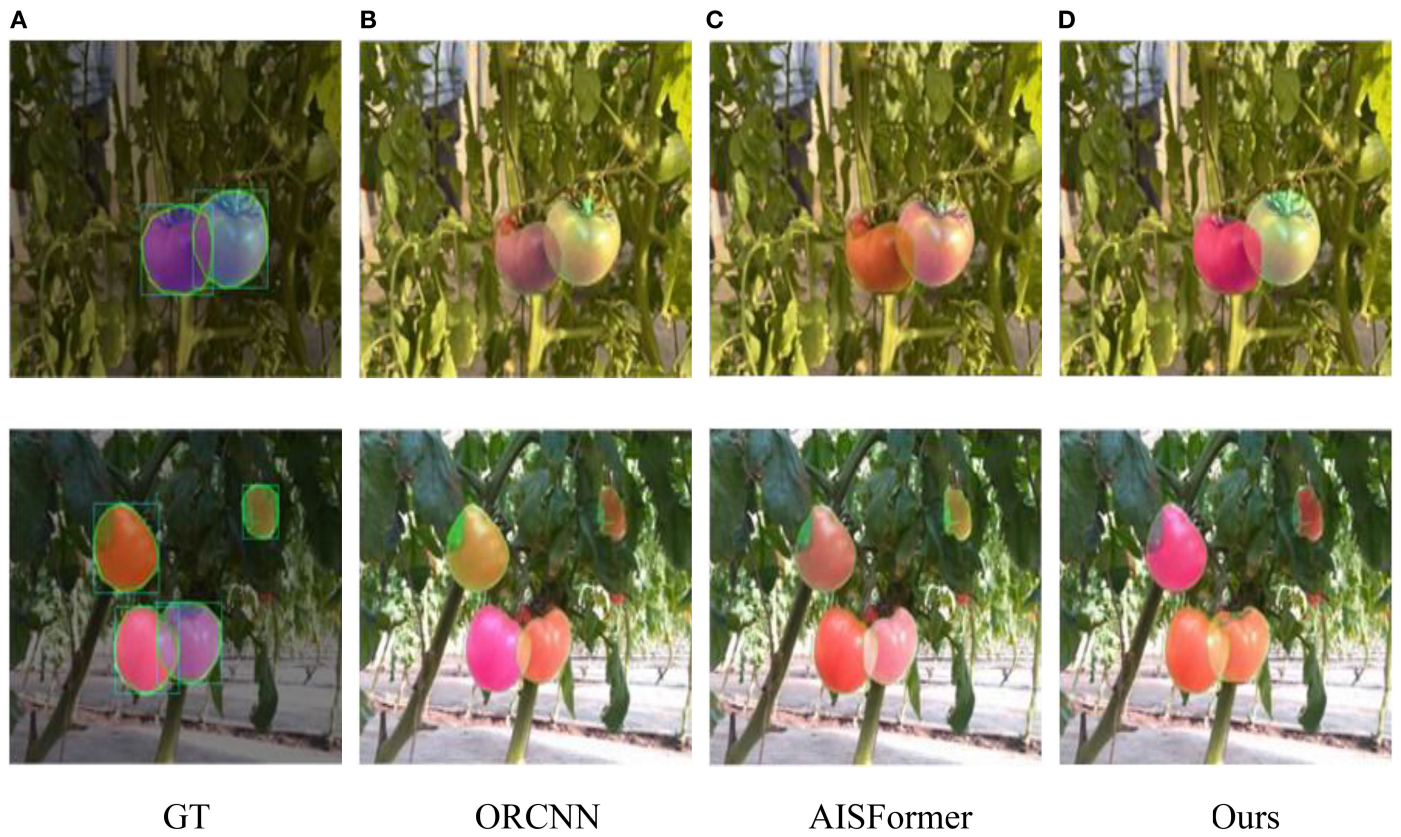
**(A–D)** Tomatoes on vines with varying color changes under different algorithms. The bottom row shows ground truth (GT), ORCNN, AISFormer, and our method, with tomatoes highlighted using bounding boxes to indicate varying ripeness and detection accuracy. Each method depicts different levels of detail and color fidelity.

Furthermore, CGA-ASNet was evaluated in a real greenhouse environment to validate its practical applicability. As shown in **Figure 15**, we selected the best- and worst-performing baseline models—ORCNN and AISFormer—for direct comparison with our method. Most results demonstrate that our model produces high-quality amodal mask predictions, with natural and consistent mask distributions across the entire ROI. In contrast, both ORCNN and AISFormer exhibit varying degrees of segmentation incompleteness or inaccuracies. Our model achieves better overall shape recovery and boundary alignment, highlighting its superior performance under real-world conditions.

### 3.7 Generalization evaluation on PApple_RGB-D-size dataset

To further assess the generalization capability of CGA-ASNet, we conducted cross-domain experiments on the PApple_RGB-D-Size dataset (Gené-Mola et al., 2023), which contains RGB-D images of apples under different illumination and occlusion conditions. This dataset significantly differs from the training domain in both fruit category, color distribution, and geometric structure, making it a suitable benchmark for evaluating robustness.

**Figure 16:**
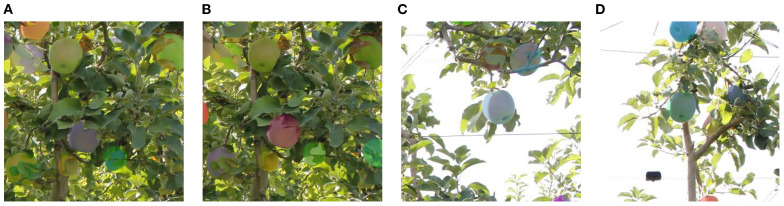
**(A)** Apples with shadows under varied lighting. **(B)** Apples with color changes under uneven illumination. **(C)** Apples in brighter light showing increased brightness. **(D)** Apples in high illumination with strong contrast.

Without any additional fine-tuning, CGA-ASNet achieved an AP50 of 89.2%, AP75 of 76.1%, and a mean Average Precision (mAP) of 73.4%, demonstrating strong generalization ability and transferability across domains. These results suggest that the model can effectively learn domain-invariant features and accurately infer the complete shape of occluded objects even under unfamiliar visual and structural conditions. In addition, **Figure 16** illustrates representative qualitative results. Despite the domain shift, CGA-ASNet is able to predict coherent amodal masks and successfully complete severely occluded fruit regions.

## 4 Conclusion

In the greenhouse environment, in order to ensure the accuracy of the non-destructive phenotype detection of tomato fruits, we constructed a virtual dataset of tomato fruits (Tomato-sim). This dataset simulated the shading conditions that occur during the actual growth of tomatoes. Additionally, for this dataset, we built an RGB-D image non-modal segmentation model based on the CGA module. We used the virtual data to train the model and then tested the model on the real data set. The following are some conclusions drawn based on the experimental results of this research work:

1. The synthetic dataset used for amodal tomato segmentation, Tomato-sim, achieved an average precision of 78.3%, closely matching the 75.0% precision obtained from real data testing. This demonstrates that synthetic data can effectively compensate for the limitations of real data collection, especially in complex agricultural scenarios, by providing flexible and diverse training conditions that handle scene complexity and object occlusion.
2. The CGA module designed in this study effectively captures the semantic information of tomatoes, particularly excelling in handling occluded regions. Compared to the baseline model, the CGA module improved the Mean Intersection over Union (mIoU) by 1.9% when dealing with occluded areas, significantly enhancing segmentation accuracy and robustness. This result further validates the CGA module’s segmentation capabilities in complex scenes, enabling better extraction of complete semantic information for partially occluded objects.

Experiments demonstrated that the CGA-ASNet model performed exceptionally well on the synthetic dataset and could effectively generalize to real greenhouse scenarios. Additionally, we tested the model on the PApple_RGB-D-Size dataset and observed similar generalization capabilities, indicating that the method is well-suited for amodal segmentation tasks involving approximately round crops like apples. The model showcased high accuracy and stability, suggesting that this approach is not limited to tomatoes but can be extended to other crops with similar shapes.

This study demonstrates that the combination of synthetic datasets and deep learning techniques provides an efficient and cost-effective solution for target segmentation in agricultural scenarios. In the future, with the expansion of dataset size and further model optimizations, the integration of synthetic and real-world data will further enhance the model’s generalization capabilities, providing robust technical support for tasks such as automated crop harvesting and crop monitoring. This also highlights the significant potential of synthetic data in agricultural vision tasks.

Despite these promising results, this study still has some limitations. First, the current method primarily focuses on crops with relatively round shapes, and its effectiveness on more complex or irregularly shaped crops remains to be validated. Second, although synthetic data improves robustness, the domain gap between synthetic and real-world data may still limit generalization in more diverse or unconstrained environments. In future work, we plan to extend our dataset to include various crop types and environmental settings, explore domain adaptation techniques, and further enhance the model’s architecture to support broader applications in agricultural perception.

## Data Availability

The original contributions presented in the study are included in the article/supplementary material. Further inquiries can be directed to the corresponding author.
